# Target-Related Alpha Attenuation in a Brain-Computer Interface Rapid Serial Visual Presentation Calibration

**DOI:** 10.3389/fnhum.2022.882557

**Published:** 2022-04-21

**Authors:** Daniel Klee, Tab Memmott, Niklas Smedemark-Margulies, Basak Celik, Deniz Erdogmus, Barry S. Oken

**Affiliations:** ^1^Department of Neurology, Oregon Health and Science University, Portland, OR, United States; ^2^Institute on Development and Disability, Oregon Health and Science University, Portland, OR, United States; ^3^Khoury College of Computer Science, Northeastern University, Boston, MA, United States; ^4^Department of Electrical and Computer Engineering, Northeastern University, Boston, MA, United States; ^5^Department of Behavioral Neuroscience, Oregon Health and Science University, Portland, OR, United States; ^6^Department of Biomedical Engineering, Oregon Health and Science University, Portland, OR, United States

**Keywords:** electroencephalography (EEG), posterior alpha, attention, brain-computer interface (BCI), event-related potential (ERP), N200, P300, signal classification

## Abstract

This study evaluated the feasibility of using occipitoparietal alpha activity to drive target/non-target classification in a brain-computer interface (BCI) for communication. EEG data were collected from 12 participants who completed BCI Rapid Serial Visual Presentation (RSVP) calibrations at two different presentation rates: 1 and 4 Hz. Attention-related changes in posterior alpha activity were compared to two event-related potentials (ERPs): N200 and P300. Machine learning approaches evaluated target/non-target classification accuracy using alpha activity. Results indicated significant alpha attenuation following target letters at both 1 and 4 Hz presentation rates, though this effect was significantly reduced in the 4 Hz condition. Target-related alpha attenuation was not correlated with coincident N200 or P300 target effects. Classification using posterior alpha activity was above chance and benefitted from individualized tuning procedures. These findings suggest that target-related posterior alpha attenuation is detectable in a BCI RSVP calibration and that this signal could be leveraged in machine learning algorithms used for RSVP or comparable attention-based BCI paradigms.

## Introduction

The development and application of brain-computer interface (BCI) technology has steadily increased over the past quarter-century (Wolpaw and Wolpaw, 2012; Rashid et al., 2020). Broadly, BCIs leverage neurophysiological signals to help users perform tasks related to movement or communication. BCI technology offers benefits to clinical populations for whom extant assistive technologies are insufficient, including individuals with locked-in syndrome (Wolpaw, 2013; Akcakaya et al., 2014). The scope, design, and neurophysiological mechanisms of BCI systems are quite varied, though BCI designs are popularly divided into two broad categories: (1) invasive systems that record data from intra-cranial electrodes, and (2) non-invasive systems that use scalp electroencephalography (EEG). Non-invasive BCI systems are more popular because they are more affordable, accessible, and do not require surgery (Akcakaya et al., 2014; Rashid et al., 2020).

Recently, software tooling in Python has been made openly available to help facilitate BCI research and development. The open-source repository BciPy (Memmott et al., 2021) includes a common BCI paradigm for communication referred to as Rapid Serial Visual Presentation (RSVP; Huang et al., 2011; Orhan et al., 2012, 2013; Acqualagna and Blankertz, 2013; Lees et al., 2018). The RSVP task presents users with a sequence of images, such as letter characters, to assess target- and non-target-related brain responses derived from scalp EEG, including the event-related potentials (ERPs) N200 and P300, which indicate stimulus discrimination and attentional processing, respectively (Patel and Azzam, 2005). RSVP may be a particularly useful paradigm in cases where users are restricted by limited eye movement or visual attention (Fried-Oken et al., 2020), since RSVP only requires a user to fixate a single stimulus presented at the center of their gaze (Acqualagna and Blankertz, 2013; Akcakaya et al., 2014).

A brain-related oscillatory signature referred to as the alpha rhythm encompasses posterior-dominant activity in the range of approximately 8–13 Hz, though the exact bounds of alpha are often debated (Bazanova and Vernon, 2014). Alpha activity is understood to result from some thalamo-cortical synchronization occurring over many centimeters, rather than any localized cortical area (Lopes da Silva et al., 1980; Lopes da Silva, 2013). Of interest to the current work, changes in alpha activity have been experimentally linked to various cortical activations. Examples of these patterns include attenuation of central alpha or mu rhythm over regions secondary to actual or imagined contralateral limb movement (Pfurtscheller et al., 2006; Meirovitch et al., 2015), or attenuation of posterior alpha with eye opening or cognitive processing of visual stimuli (Klimesch, 1999; Pfurtscheller and Lopes da Silva, 1999). Additionally, changes in the distribution of posterior alpha are known to track shifts in covert spatial attention (Foster et al., 2017).

Prior research has explored the relationship between attention-related changes in alpha and ERPs. Some evidence has suggested that alpha desynchronization and target-related P300 may index and predict similar attentional processes (Yordanova et al., 2001; Grent-’T-Jong et al., 2011), or that evoked potentials have the potential to interfere when measuring attention-related changes in alpha activity (van Gerven et al., 2009). However, it is known that visual attention can modulate alpha amplitude in the absence of ERP signals (Klimesch, 1999), and source analysis has associated these signals with different cortical structures (Peng et al., 2012).

A few studies have demonstrated the feasibility of using posterior alpha to track spatial attention in order to make letter selections in BCIs for communication (Kelly et al., 2005; van Gerven et al., 2009; van Gerven and Jensen, 2009; Treder et al., 2011). Evidence suggests that posterior alpha remains a viable signal for classification over repeated sessions (Horschig et al., 2015), and that patterns of alpha lateralization can be altered through neurofeedback training (Okazaki et al., 2015). In addition to spatial attention, posterior alpha might also be used to track a user’s mental state during BCI tasks (Myrden and Chau, 2017). However, despite being widely studied and relatively easy to measure, vision-modulated posterior alpha is neither a popular nor a common component of many BCI spellers (Rezeika et al., 2018; Rashid et al., 2020). To our knowledge, no previous work has attempted to use attention-related changes in posterior alpha to make letter selections in the context of a centrally fixated BCI spelling paradigm such as RSVP.

Given the evidence that visual attention modulates posterior alpha activity, and the conspicuous absence of posterior alpha as a driving neural signal in non-invasive BCI spelling systems, we conducted an exploratory study to examine the feasibility of using this signal in the context of the BciPy RSVP paradigm. A novel contribution of the current study is that no prior BCI-related work has examined posterior alpha changes outside of lateralized displays. Our primary aims were to determine whether target-related changes in posterior alpha activity are detectable in the RSVP task and, if so, whether these changes are sensitive to the presentation rate of letter stimuli. We hypothesized: (1) that event-related alpha activity would decrease following the presentation of target letter stimuli relative to non-target stimuli; and (2) that target-related posterior alpha attenuation would be smaller at an increased rate of presentation due to temporal overlap of target processing with subsequent non-targets. As secondary aims, we sought to assess whether target-related posterior alpha effects correlated with coincident target effects of N200 and P300 ERPs, and whether we could train machine learning algorithms to classify target and non-target responses using posterior alpha signals alone.

## Materials and Methods

EEG data were recorded from a convenience sample of generally healthy adults recruited at Oregon Health and Science University (OHSU) in Portland, OR. Research activities were registered with and approved by the OHSU Institutional Review Board (IRB). Data were collected over the course of a single 90-min session, after which participants were compensated $25 for completing the study.

### Participants

Demographic information is presented in Table 1. Twelve generally healthy adults enrolled in the study and provided written informed consent before participating in study activities, in accordance with the Declaration of Helsinki. All individuals recruited for the study were fluent in English. No participants reported use of alcohol or other mind-altering substances within 12 h of their test session. Exclusion criteria included use of EEG-altering medications such as neuroleptics or benzodiazepines (as reviewed by a physician), or an inability to perceive RSVP task stimuli and achieve at least 80% accuracy on a RSVP practice task. No one who enrolled was ineligible to participate based on these criteria. All participants were confirmed to have normal or corrected-to-normal vision and a minimum near-field Snellen visual acuity of 20/30 in at least one eye. All participants achieved perfect scores on the practice RSVP task after only a single attempt.

**TABLE 1:** Demographics.

	Participants (*n* = 12)
**Age:** mean years ± SD (range)	33.75 ± 6.40 (28–46)
**Sex**	
Female	6
Male	6
**Race**	
White	12
**Ethnicity**	
Hispanic/Latino	1
Not-Hispanic/Latino	11

*A summary of participants enrolled in the study. Our sample contained a balanced number of female and male participants, but was homogeneous with regard to race and ethnicity.*

### Procedure

After providing written informed consent at the start of the study visit, participants self-reported demographic information followed by a brief health questionnaire and near-field visual acuity test. Upon completion of the screening questions, participants were introduced to a practice version of the RSVP calibration (see section “Rapid Serial Visual Presentation Practice”) before donning the EEG cap. Participants then completed two instances of RSVP calibration (see section “Rapid Serial Visual Presentation Calibration”). Individuals were asked to indicate how sleepy they felt immediately before, between, and after the two calibrations using the Stanford Sleepiness Scale (SSS; Herscovitch and Broughton, 1981; MacLean et al., 1992), since research has demonstrated that alpha activity fluctuates during periods of drowsiness (Cantero et al., 2002).

### Screening

#### Self-Report Questionnaires

For screening purposes, individuals were asked to self-report current health conditions, medications, and provide details about their sleep habits. Specifically, participants were asked to indicate whether they had problems sleeping too much or too little, or if they had seen a physician for a sleep-related disorder.

#### Rapid Serial Visual Presentation Practice

To confirm participants’ ability to perceive the RSVP stimuli (see section “Rapid Serial Visual Presentation Calibration”), lab staff administered a practice version of the RSVP calibration created in PsychoPy3 Experiment Builder, version 3.0.0b11 (Peirce, 2007, 2009). Unlike the test version of RSVP calibration, the practice task presented only 10 trials of pseudo-random letter sequences, each of which had a 50% chance to contain the target letter. Stimuli in the letter sequences appeared at a rate of 4 Hz in order to emulate the more difficult experimental condition. Participants were asked after each 10-letter sequence to state whether the target had been present.

### Rapid Serial Visual Presentation Calibration

#### Overview

**FIGURE 1:**
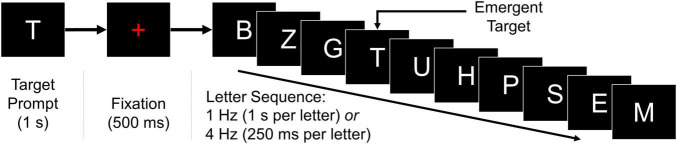
RSVP calibration. Each instance of the RSVP calibration consisted of 100 ten-letter sequences, as illustrated. A target letter was presented on-screen for 1 s, followed by a red fixation cross for 500 ms, and then a random letter sequence containing the target. The 10-letter sequences were presented at a rate of either 1 or 4 Hz for the entirety of the task. A blank inter-stimulus interval of 750 ms separated the final letter of each sequence and the target prompt of the subsequent trial.

Participants completed two instances of the BciPy RSVP calibration (Orhan et al., 2011, 2012, 2013; Oken et al., 2014; Memmott et al., 2021). Each calibration consisted of 100 trials of 10-letter sequences and lasted approximately 10–25 min, depending on the presentation rate of letter stimuli. As demonstrated in Figure 1, each trial began with a target letter prompt presented for 1 s, followed immediately by the onset of a red fixation cross for 500 ms, and then a steady-stream sequence of 10 letter stimuli presented either at a rate of 1 or 4 Hz (1.0 s or 250 ms per stimulus, respectively). There were no blank-screen intervals within each sequence; a blank black screen was presented for 750 ms between sequences.

Participants were instructed to sit still, try to blink only between sequences, and watch for the target letter in each sequence, as indicated by the most recent target prompt. When the target appeared, participants were told to react mentally without movement or blinking. In cases where an individual was unable to resist blinking during the sequence (e.g., due to fatigue or irritation), there was a standing instruction to prioritize blinking during a non-target presentation in order to avoid missing the target. Along these lines, participants were allowed to pause the task by pushing the spacebar if they needed to rest.

**FIGURE 2:**
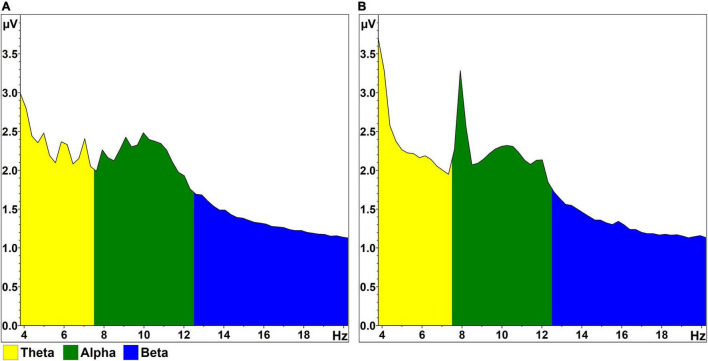
RSVP FFT output. **(A)** Mean FFT magnitude (normalized amplitude; pooled occipitoparietal sites), averaged across all 1,000 stimulus epochs in the 1 Hz condition and across all participants. A peak is visible at 10 Hz, within the range of alpha activity. **(B)** Similar FFT data, generated from the 4 Hz condition. Again, a peak is visible at 10 Hz, bookended by sharper peaks reflective of the 2nd (8 Hz) and 3rd (12 Hz) harmonics of the SSVEP, which results from high-contrast flickering (4 Hz) in the letter stream.

Each participant completed RSVP calibrations with stimuli presented at rates of 1 Hz and 4 Hz; these rates were consistent within each 100-trial instance of the task. Condition order was balanced randomly across the 12 participant sessions in order to minimize systematic fatigue effects associated with repetition of the RSVP task (Oken et al., 2018). The logic behind our use of 1 and 4 Hz presentation rates was twofold: (1) to present stimuli at speeds where early visual and attentional processing of sequential stimuli would both overlap (i.e., 4 Hz; 250 ms per letter stimulus) and not overlap (i.e., 1 Hz; 1 s per letter stimulus); and (2) to minimize contamination of alpha-band activity by harmonics related to steady-state visual-evoked potential (SSVEP) artifact. Onset of the N200/P300 complex is expected 200–300 ms post-stimulus onset (Patel and Azzam, 2005), while target-induced posterior alpha desynchronization effects have been demonstrated approximately 300–800 ms following stimulus onset in a visual oddball paradigm (Peng et al., 2012). Past work with this RSVP paradigm has typically used a presentation rate of 5 Hz (e.g., Oken et al., 2018). However, as shown in Figure 2, the 2nd and 3rd harmonics of the SSVEP are sometimes apparent following spectral decomposition. To avoid contamination from a 10 Hz harmonic in the middle of the alpha band, the “typical” RSVP presentation rate of 5 Hz was lowered to 4 Hz.

#### Stimuli

RSVP stimuli were rendered on a 17.3-inch ASUS Vivobook Pro N705F laptop (1,920 × 1,080 resolution) with a refresh rate of 60 Hz. Participants viewed the display from a seated position in a dimly lit room with consistent lighting. Viewing distance was approximately 70 cm, though head position was not constrained. Fixation crosses were drawn in solid red. Letter stimuli were drawn in sans-serif Arial font and rendered solid white against an unchanging solid black background. Letter stimuli included 28 possible characters: capitalized forms of all 26 letters of the English alphabet, plus the characters “_” and “<,” which represent “space” and “backspace” in this RSVP paradigm, respectively.

At a viewing distance of 70 cm, the red fixation cross subtended 2.5^°^ visual angle with a stroke width of 0.4^°^. All letter stimuli had a stroke width of approximately 0.5^°^. Due to morphological differences in the 28 letter stimuli, character height ranged between 0.5° (i.e., “_”) and 3.8° (e.g., “T”). Similarly, letter width ranged between 0.5° (i.e., “I”) and 4.8° (i.e., “W”).

### Electrophysiological Recordings

EEG data were recorded from a DSI VR-300 headset (Wearable Sensing; San Diego, CA, United States). This adjustable dry-electrode system utilized a linked-ear reference with a ground at A1. The sampling frequency was 300 Hz, with an A/D resolution of 16 bits. Electrodes were placed according to a custom montage of standard 10/20 sites: FCz, F7, Pz, P4, PO7, PO8, and Oz. These sites are optimized to capture P300 evoked potentials (Krusienski et al., 2008), with the alteration that site P3 was replaced by F7 as an additional anterior site in order to capture eye-related activity (e.g., blinking). Signal quality was assessed in Wearable Sensing’s DSI-Streamer software (v.1.08.44) prior to completion of the experimental tasks. Electrodes were adjusted to satisfy manufacturer-determined default operating thresholds pertaining to clipping, noise (very low < 0.07 μVrms; very high > 100 μVrms), impedance (<2.0 MΩ), impedance signal-to-noise (<3.0 MΩ), and DC offset (50% of maximum). All experimental EEG data were recorded using the BciPy acquisition client.

### Electrophysiological Processing

With the exception of the secondary BCI classification comparisons (see section “Brain-Computer Interface Classifiers”), all time-frequency and ERP analyses were performed offline in BrainVision Analyzer: Professional Edition, version 2.1.0.327 (BrainVision LLC; Morrisville, NC, United States). Due to *a priori* uncertainty regarding the exact distribution of the attention effect across the scalp during RSVP calibration, EEG measures were analyzed twice in parallel: both as a “pooled” (i.e., averaged) signal across occipitoparietal sites Pz, Oz, PO7, and PO8, and also at site Pz in isolation (i.e., “Pz-only”). Pz is typically where experimenters can expect to observe the highest-amplitude target-related P300 (Polich, 2007), and was therefore selected as a representative sub-site. Data were filtered 1–45 Hz (48 dB/octave) along with a 60 Hz notch filter using a Butterworth zero-phase infinite impulse response (IIR) filter. These filtered data were then downsampled from 300 to 150 Hz.

#### Time-Frequency Analyses

EEG data in the time-frequency analysis were segmented into 2.5 s epochs centered relative to stimulus onset, such that each of the 100 target and 900 non-target epochs in a given calibration ranged from −1,250 ms to +1,250 ms relative to stimulus onset. This window was chosen in order to capture activity ±1 s relative to stimulus onset, with ample buffer length of 250 ms at either end of the window to avoid edge-related artifact due to the wavelet analysis. Within each calibration recording, epochs were separated by condition and converted to time-frequency scaleograms using a continuous wavelet transform (CWT) with a complex-valued Morlet mother wavelet (Morlet parameter *c* = 5). Scaleograms were generated from wavelets with scaled frequencies ranging from 4 to 16 Hz in 48 logarithmic steps, normalized according to uniform scale power (unit energy normalization; all scaled frequency layers of the wavelet function possessed an energy value of 1). Complex voltage output was converted to real-valued voltage (μV). The mean and standard deviation of a 500 ms baseline window ranging from −600 to −100 ms before stimulus onset were used to Z-transform all output samples within the test epochs. Unless otherwise specified, “alpha activity” in this analysis refers to the average of the Z-scored real voltage values within the designated response window of 300–800 ms post-stimulus onset, which was selected to capture the onset and duration of the target effect as seen in previous research with visual oddball paradigms (Peng et al., 2012; Vázquez-Marrufo et al., 2019). In other words, “negative” activity values indicate event-related decreases in alpha activity relative to the baseline window (−600 to −100 ms). See BrainVision Analyzer User Manual (Software Version 2.1.0) for further information regarding operations (Brain Products, 2014).

Our analyses examined alpha activity following target and non-target letter stimuli, as well as the alpha attenuation effect, which we defined as the difference between target and non-target alpha responses within each RSVP calibration. Statistical tests were performed on alpha activity estimates taken from the single wavelet layer with scaled central frequency closest to each participant’s individual alpha frequency (IAF) within a calibration, rounded to the nearest 0.5 Hz. Identifying spectral prominences prior to time-frequency investigations is a recommended technique to avoid misleading analyses of arbitrary noise (Pfurtscheller and Lopes da Silva, 1999; Corcoran et al., 2018). IAF estimates were determined from mean fast Fourier transformation (FFT) output from the pooled occipitoparietal signals; this FFT output was averaged across all available 2.5 s epochs within each individual instance of RSVP calibration (Figure 2). Real-valued voltage output was generated using a 20% Hanning window, periodic variance correction, and amplitude normalization relative to a frequency bandwidth of 4–20 Hz. Semi-automatic peak detection identified the frequency in the range of 7.5–12.5 Hz with the highest magnitude. Estimates were then reviewed visually and adjusted in the event of peak capture by either noise or SSVEP artifact in the 4 Hz presentation condition. Clear alpha peaks were absent in two of the 1 Hz calibrations and three of the 4 Hz calibrations. In these cases where no discernable peaks were present, the peak estimate was set manually to 10 Hz. The underlying rationale of this approach was that a wavelet layer with scaled central frequency of 10 Hz and spectral bandwidth of approximately 4 Hz is well-positioned to capture most of the signal within the alpha band (8–12 Hz). Peak values determined in this manner ranged from 9 to 11.5 Hz across participants; all participants demonstrated similar IAF estimates between the 1 and 4 Hz presentation conditions, with the exception of three individuals who showed marginal increases in peak alpha of 0.5 Hz (two instances) and 1.0 Hz (one instance) in the 4 Hz condition.

#### Event-Related Potential Analyses

To accommodate ERP analyses, the original data were segmented into 1 s epochs ranging from −200 to +800 ms, relative to stimulus onset. These epochs were baseline corrected (−200 to 0 ms), separated by stimulus-type, and averaged within-condition before the use of semi-automatic peak detection to label N200 (200–350 ms) and P300 (300–450 ms) potentials in the target condition. N200 and P300 responses within both the target and non-target classes were estimated as mean voltage (μV) ± 4 sampled points (∼53 ms) around the labeled peak latencies derived from the target samples. Our analyses measured N200 and P300 signed voltages following target and non-target letter stimuli, as well as the target effect, which we defined as the difference between target and non-target voltage responses within each condition.

#### Artifact Rejection

To approximate the processing tools included by default in BciPy, which currently does not include tooling for artifact handling outside of filtering, our primary offline analyses of EEG and ERP data did not implement rigorous artifact rejection procedures. However, to ensure that our results were not influenced by poor data quality and that electrooculographic (EOG) activity had no effect on our alpha measurements, we re-ran key analyses using data that included artifact rejection to remove eye blinks, extraneous electromyography (EMG), and other non-descript noise such as electrode popping due to movement and poor electrode contact. All time-frequency and ERP epochs were flagged for review and semi-automatic rejection according to the following rules: voltage gradients > 50 μV/ms; voltage differences > 125 μV within a window of 50 ms; absolute voltage values > 75 μV; and activity < 0.5 μV sustained for ≥ 100 ms in duration. Data marked in this manner were reviewed visually and contaminated epochs were discarded prior to separating epochs by stimulus class (i.e., target vs. non-target).

Artifact rejection results are discussed under “Results” section “Artifact Rejection.” Individual participants kept at least 70% of target time-frequency epochs in the 1 Hz condition and 65% of targets at 4 Hz, with the exceptions that one participant only maintained 59 target segments in the 1 Hz condition, and another kept only 46 target segments in the 4 Hz condition. Across ERP epochs, individual participants kept ≥85% of target segments at 1 Hz and ≥78% of targets at 4 Hz.

### Brain-Computer Interface Classifiers

The Python code underlying our BCI classifier analyses is accessible online (Zenodo, 2022).

#### Brain-Computer Interface Classifiers: Alpha Data

For the purpose of training classifiers using alpha-band features, we performed analogous time-frequency preprocessing of the raw EEG data using the PyWavelets Python package (Lee et al., 2019). Due to differences in software, the wavelet parameters used here differed slightly from those outlined above in section “Time-Frequency Analyses”: we used a Morlet mother wavelet with bandwidth of 1.5 Hz and a central frequency of 1.0 Hz; scaled wavelets were not normalized. Data were Z-scored according to the same 500 ms baseline (−600 to −100 ms) and response (+300 to +800 ms) windows, and we selected one frequency to investigate for each participant (see IAF in “Materials and Methods” section “Time-Frequency Analyses”). Unlike the primary analysis, however, alpha activity was preserved as time-series data and was not averaged within the response window. We also experimented with optimizing the locations of these 500 ms baseline and response windows to improve classification. We varied the baseline window start between −1,050 and −600 ms, and varied the response window start between +150 and +550 ms, selecting the best starting positions according to the balanced test accuracy of a Logistic Regression classifier. Classifier analyses were conducted using data from all four of the occipitoparietal channels.

We evaluated several classifiers on this time-series representation of the alpha data, including Logistic Regression with L2 regularization and a support vector classifier. We also tried classifying these data in a channelwise covariance matrix representation, using a logistic regression classifier on a tangent space projection (Barachant et al., 2012, 2013). The experimental setup results in a fixed class ratio of 9 non-targets for every target; thus, all models were trained using class-balanced objectives and evaluated using balanced accuracy, which is the average of positive (target) and negative (non-target) class accuracy: *acc*_*bal*_=(*acc*_*target*_+*acc*_*non*−*target*_)/2. Note that a model that makes uniform random guesses will achieve a balanced accuracy of approximately 0.5 (i.e., at chance levels), while a perfect model will achieve an accuracy of 1.0.

#### Brain-Computer Interface Classifiers: Event-Related Potential Data

To compare alpha classification performance to the default classification mechanism used in BciPy, we used principal component analysis, followed by regularized discriminant analysis and kernel density estimation (RDA/KDE) to classify filtered EEG time-series data inclusive of the N200 and P300 ERPs. This approach has been detailed in previous work (Oken et al., 2018; Memmott et al., 2021) and was used to generate balanced accuracy estimates similar to those of the alpha classifiers outlined in section “Brain-Computer Interface Classifiers: Alpha Data.” Unlike the ERP analyses in “Materials and Methods” section “Event-Related Potential Analyses,” ERP time-series data were filtered 2–45 Hz and segmented into 500 ms epochs ranging 0 to +500 ms relative to stimulus onset in order to match the default settings of BciPy.

### Statistical Analyses

Statistical tests were conducted in IBM SPSS Statistics, Version 27 (IBM Corporation; Armonk, NY, United States). Shapiro-Wilk tests were used to assess the normality of our measures; box plots were reviewed to identify relevant outliers. Roughly one-third of the primary across-participant alpha and ERP measures were not normally distributed, trending (*p* < 0.10) toward non-normality, or contained at least one outlier. Because these measures were inconsistently normally distributed, we opted to forego parametric means comparisons and instead conducted across-participant median comparisons using non-parametric two-tailed related-samples Wilcoxon signed rank tests. Shapiro-Wilk tests also revealed that most of the within-participant alpha measures were not normally distributed, so independent-samples Mann-Whitney *U*-tests were used to evaluate target vs. non-target alpha activity differences within individual RSVP calibrations. These data were inspected visually to confirm general similarity of shape. Spearman’s rank-order correlations were used to examine the relationships between alpha attenuation effects and ERP target effects. Similarly, artifact rejection and tuned parameter analyses used non-parametric tests to accommodate non-normal data and outliers.

## Results

Self-reported sleepiness levels demonstrated a significant median increase between the start (*Mdn* = 3.0) and mid-point (*Mdn* = 3.5) of the experiment, *Z* = −2.111, *p* = 0.035. However, there were no significant median differences in sleepiness levels before RSVP, after RSVP, or sleepiness changes after-minus-before RSVP according to condition, either 1 or 4 Hz presentations (all *p* values ≥ 0.165). None of the self-reported SSS sleepiness metrics correlated with any of the alpha attenuation effects [all absolute *r*_*s*_(10) values ≤ 0.511; all *p* values ≥ 0.09].

### Time-Frequency Analyses

**FIGURE 3:**
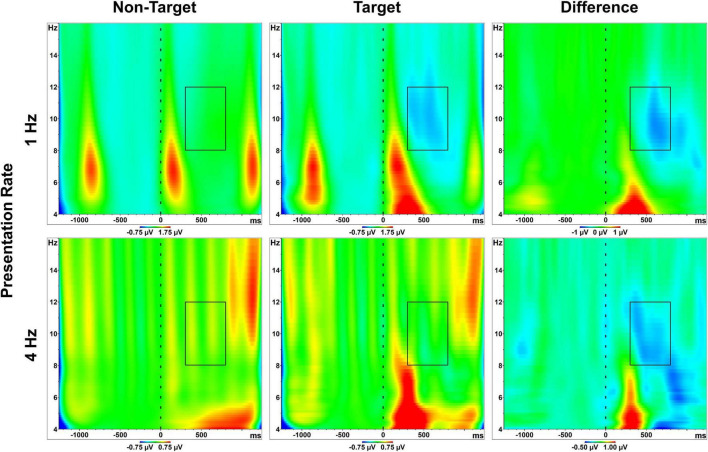
Scaleograms. Spectra 4–16 Hz, relative to stimulus onset (*t* = 0). Plots are derived from pooled posterior sites, averaged across all participants, and separated by condition (presentation rate; stimulus class). Boxes represent the alpha band (8–12 Hz) and the measured effect window. The final column illustrates the alpha attenuation effect, or the difference in activity between target and non-target letter responses. SSVEP signatures are visible across conditions, both as conspicuous bursts in the range of 5–9 Hz (the 1 Hz presentation condition), and as more spectrally-diffuse vertical stripes (the 4 Hz presentation condition). A clear increase in theta activity is also visible approximately 200–300 ms following target stimuli. Color scaling is consistent between targets/non-targets within presentation rate conditions. However, due to gross differences in alpha activity between the 1 Hz and 4 Hz conditions (see Figure 4, which includes static scaling of axes across conditions), scaling was adjusted to better highlight the time-course of the attenuation effect.

Time-frequency scaleograms displayed in Figure 3 show representative wavelet output 4–16 Hz during the RSVP calibrations. As clarified previously in section “Artifact Rejection,” these primary analyses included all 1,000 available epochs (both target and non-target) from all 100 trials in each RSVP calibration. Artifact rejection comparisons and the corresponding redundant analyses are discussed separately below in “Results” section “Artifact Rejection.”

#### Alpha Effects: Across-Participants

**FIGURE 4:**
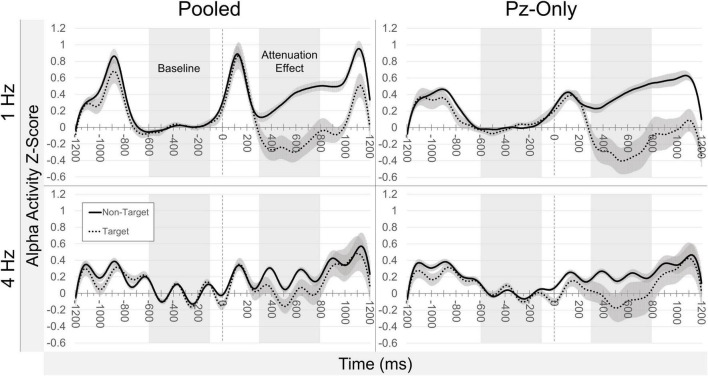
Grand average alpha activity. Waveforms illustrate continuous alpha activity (Z-score) averaged across all participants (±SEM), relative to stimulus onset (*t* = 0). Plots are separated by condition (presentation rate; stimulus class) and electrode source (pooled occipitoparietal sites; Pz-only). Highlighted regions of the waveforms are indicative of baseline and the measured effect windows.

Figure 4 offers a summary of Z-scored alpha activity waveforms across participants (± standard error of the mean; SEM) according to electrode source, presentation rate, and stimulus class. Measuring the pooled occipitoparietal signal in the 1 Hz condition, target-related median alpha activity (*Mdn* = −0.164) was significantly lower than for non-target letters (*Mdn* = 0.285), *Z* = −2.667, *p* = 0.008. Similarly, in the 4 Hz condition, median alpha activity was lower following targets (*Mdn* = −0.022) compared to non-targets (*Mdn* = 0.172), but this difference did not reach statistical significance, *Z* = −1.883, *p* = 0.060. The size of the target-vs.-non-target attenuation effect was significantly larger at 1 Hz presentation rates (*Mdn* = −0.632) compared to 4 Hz presentation rates (*Mdn* = −0.227), *Z* = −2.353, *p* = 0.019.

Using data from Pz-only, we observed effects similar to those in the pooled signal. In the 1 Hz condition, median alpha activity for targets (*Mdn* = −0.103) was significantly lower than median alpha related to non-targets (*Mdn* = 0.373), *Z* = −3.059, *p* = 0.002. At 4 Hz presentations, median target-related alpha activity (*Mdn* = 0.098) was again lower than that of the non-target stimuli (*Mdn* = 0.196), though unlike data from the pooled signal, this difference was significant at Pz-only, *Z* = −1.961, *p* = 0.050. The median alpha attenuation effect at Pz-only was larger in the 1 Hz condition (*Mdn* = −0.527) compared to the 4 Hz condition (*Mdn* = −0.103), *Z* = −2.510, *p* = 0.012. There were no meaningful differences between median alpha attenuation in the pooled signal and at Pz-only, either at 1 or at 4 Hz presentations (both comparisons: *Z* = −0.784, *p* = 0.433).

#### Alpha Effects: Within-Participants

**FIGURE 5:**
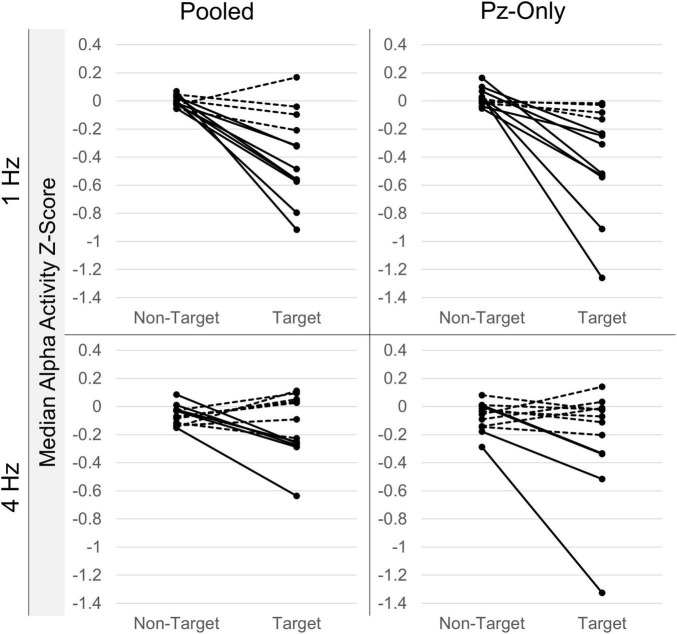
Intra-individual alpha attenuation effects. Median target and non-target alpha activity Z-scores from individual RSVP calibrations. Data are sorted according to presentation rate and electrode source. Lines connect corresponding non-target and target estimates from within-individual recordings. Significant within-participant non-target/target median differences (*p* < 0.05; Mann-Whitney *U*-tests) are denoted by solid lines; non-significant differences (*p* > 0.05) are drawn as dashed lines. Plots show clear variability in the attenuation effect across individual participants and presentation conditions. Alpha attenuation effects appear to be greater at Pz for select individuals, but also more variable across participants.

While between-participant effects are of interest, the use of alpha signals within BCI systems ultimately requires significant within-participant effects. Independent-samples Mann-Whitney *U* comparisons revealed significant median differences between target and non-target alpha Z-scores within individual RSVP calibrations. Significant attenuation of alpha activity following target letter stimuli was discernable in at least one of the signal sources (i.e., either the pooled signal or Pz-only) in 9/12 participants in the 1 Hz condition, and 6/12 participants in the 4 Hz condition (see Figure 5 for a summary of within-participant alpha attenuation effects). The attenuation effect was equally detectable between the pooled occipitoparietal signal and Pz-only during 1 Hz presentations, such that 7 participants showed significant median differences between target and non-target alpha activity in both signal sources, while 1 participant evinced the attenuation effect only in the pooled signal and another just at Pz-only. This pattern was comparable in the 4 Hz condition, where 3 participants demonstrated the effect in both signals, 2 showed changes only in the pooled signal, and 1 just at Pz-only.

### Event-Related Potential Analyses

**FIGURE 6:**
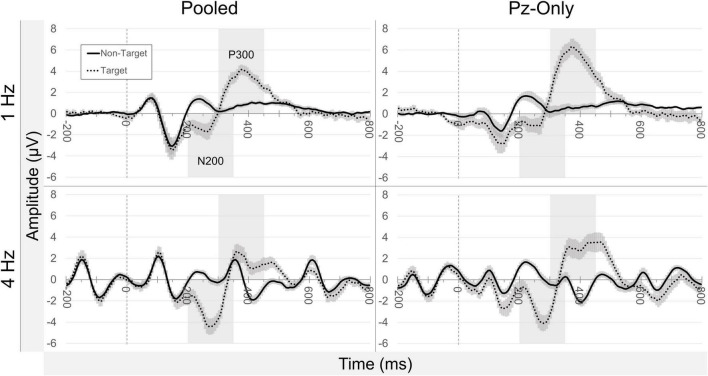
Grand average ERP waveforms. Grand average ERP waveforms averaged across all participants (±SEM), relative to stimulus onset (*t* = 0). Plots are separated by condition (presentation rate; stimulus class) and electrode source (pooled occipitoparietal sites; Pz-only). Highlighted temporal regions illustrate default window settings of peak detection for the N200 and P300 potentials.

Many of the expected ERP target effects were observed across pooled occipitoparietal sites (Figure 6). N200 and P300 measures were all increased following target stimuli compared to non-target stimuli, both at 1 and 4 Hz presentation rates (all *p* values ≤ 0.012). The size of the N200 target effect was smaller in the 1 Hz condition (*Mdn* = −1.629) compared to the 4 Hz condition (*Mdn* = −3.842), *Z* = −2.746, *p* = 0.006, while the corresponding P300 target effect was inversely greater in the 1 Hz condition (*Mdn* = 3.262) than in the 4 Hz condition (*Mdn* = 1.508), *Z* = −2.435, *p* = 0.015. These ERP target effects remained unchanged when measured at Pz-only, with increased N200 and P300 measures for targets compared to non-targets (all *p* values ≤ 0.003). The median N200 target effect was larger during 4 Hz presentations (*Mdn* = −3.512) compared to 1 Hz presentations (*Mdn* = −2.648), *Z* = −2.746, *p* = 0.006, and the median P300 target effect was again larger at 1 Hz (*Mdn* = 5.513) compared to the 4 Hz condition (*Mdn* = 3.501), *Z* = −2.118, *p* = 0.034. N200 target effects were not discernably different between the pooled signals and Pz-only in either presentation condition (both *p-*values ≥ 0.433). P300 target effects were larger at Pz-only than in the pooled signal in both the 1 and 4 Hz conditions (both *p* values = 0.002).

### Correlation of Across-Participant Alpha Attenuation and Event-Related Potential Target Effects

**FIGURE 7:**
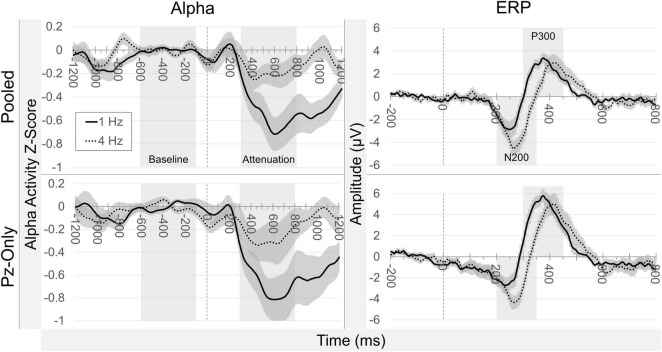
Difference waveforms. Grand average difference waveforms illustrate target minus non-target signals. Alpha attenuation (column 1) and ERP target effects (column 2) are separated according to signal source and presentation rate condition. Highlighted temporal windows correspond to baselines and measured attenuation (alpha), and default peak detection settings (ERPs). There is a clear reduction in alpha attenuation in the 4 Hz condition, while N200 and P300 target effects remain relatively similar in magnitude between presentation rates.

Alpha attenuation and ERP target effects are depicted side-by-side as difference waveforms in Figure 7. Across pooled occipitoparietal sites in the 1 Hz condition, alpha attenuation effects did not correlate with N200 [*r*_*s*_(10) = −0.133, *p* = 0.681] or P300 target effects [*r*_*s*_(10) = −0.105, *p* = 0.746]. Pooled signal alpha attenuation effects in the 4 Hz condition did not meaningfully predict N200 [*r*_*s*_(10) = −0.329, *p* = 0.297] or P300 target effects [*r*_*s*_(10) = −0.007, *p* = 0.983]. This disconnect between alpha and ERP target effects in the pooled occipitoparietal signal was also evident in the Pz-only signal. As before, in the 1 Hz condition there were no significant correlations between alpha attenuation and ERP target effects for N200 [*r*_*s*_(10) = 0.084, *p* = 0.795] or P300 [*r*_*s*_(10) = 0.035, *p* = 0.914]. At 4 Hz, the Pz-only signal did not yield any significant correlations between alpha attenuation and target effects for either N200 [*r*_*s*_(10) = −0.042, *p* = 0.897] or P300 [*r*_*s*_(10) = 0.000, *p* = 1.000].

### Artifact Rejection

Related-sample Wilcoxon signed ranks tests indicated that none of the between-participant alpha measures were statistically different before and after artifact rejection, as outlined in “Materials and Methods” section “Artifact Rejection” (all *p* values > 0.05). The results of our alpha comparisons remained unchanged, with a single exception that the difference between median target and non-target alpha activity at Pz-only was no longer statistically different at 4 Hz presentations (*p* = 0.308). Spearman’s rank-order correlations indicated no significant correlations between alpha attenuation and ERP target effects in either the pooled [all absolute *r*_*s*_(10) values ≤ 0.476; all *p* values ≥ 0.118] or Pz-only signals [all absolute *r*_*s*_(10) values ≤ 0.238; all *p* values ≥ 0.457].

Within-participants, artifact rejection did not result in visible changes to the shape of alpha activity distributions; the majority of measures remained non-normal, including at least one class (i.e., target or non-target) within each electrode source per individual RSVP calibration. Artifact rejection resulted in changes to the alpha attenuation effect primarily in the 4 Hz condition, where 3 previously significant effects dissipated and 1 moved from significant (*p* < 0.05) to trending (*p* < 0.10). In the 1 Hz condition as well, 1 previously significant effect shifted to trending while another dissipated entirely.

### Implementation and Classification

#### Regularized Discriminant Analysis and Kernel Density Estimation

Mean balanced test accuracies from the ERP RDA/KDE model ranged 0.595–0.836 across participants in the 1 Hz condition and 0.614–0.874 in the 4 Hz condition. A paired-samples Wilcoxon test indicated a significant median increase in test accuracies between the 1 Hz condition (*Mdn* = 0.676) and the 4 Hz condition (*Mdn* = 0.707), *Z* = −2.433, *p* = 0.015. Within the 1 Hz condition, there was a significant Spearman’s correlation between mean balanced test accuracies of the RDA/KDE model and N200 target effects [*r*_*s*_(10) = −0.776, *p* = 0.003]. The same RDA/KDE estimates related to P300 target effects [*r*_*s*_(10) = 0.531, *p* = 0.075], but only weakly. 1 Hz condition RDA/KDE accuracies were not correlated with alpha attenuation [*r*_*s*_(10) = 0.287, *p* = 0.366]. At 4 Hz presentations, mean balanced test accuracies from the RDA/KDE model were associated with N200 [*r*_*s*_(10) = −0.790, *p* = 0.002] and P300 target effects [*r*_*s*_(10) = 0.608, *p* = 0.036], though RDA/KDE accuracies were again unrelated to alpha attenuation effects [*r*_*s*_(10) = 0.077, *p* = 0.812].

#### Alpha Classifiers

**FIGURE 8:**
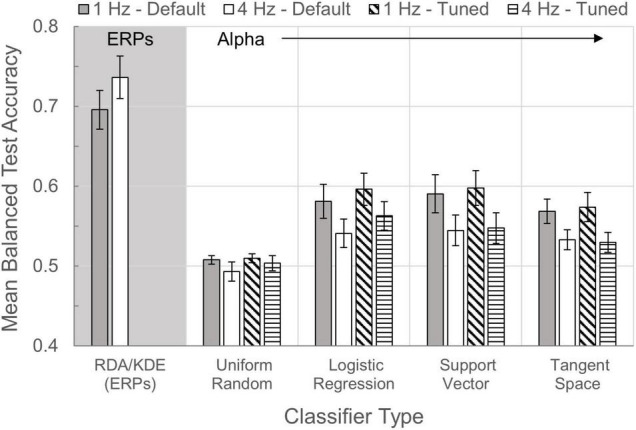
Classifier comparisons. Bars illustrate the mean balanced test accuracies (±SEM) of multiple classifiers in the 1 and 4 Hz presentation conditions. With the exception of RDA/KDE, which quantifies performance of the ERP time-series classification, all other classifiers examine time-series alpha activity. The Uniform Random model was used as a control classifier with an expected classification accuracy of 0.50.

A comparative bar chart of the various alpha and ERP classifier methods is shown in Figure 8. Default parameters yielded mean balanced test accuracies for individual participants that ranged 0.470–0.764 across models in the 1 Hz condition and 0.463–0.729 in the 4 Hz condition. Tuned parameters resulted in mean balanced test accuracies ranging 0.508–0.772 across models in the 1 Hz condition and 0.472–0.732 in the 4 Hz condition. Across participants at both 1 and 4 Hz presentations, both with default and tuned parameters, all alpha modeling underperformed relative to accuracy estimates from the ERP RDA/KDE model (Wilcoxon: all *p* values ≤ 0.008). Alpha attenuation effects were significantly correlated with all default classifier mean test accuracy estimates at 1 Hz (*p* values ≤ 0.039), with the expected exception that there was no meaningful correlation between alpha attenuation and the Uniform Random model. In contrast, alpha attenuation in the 4 Hz condition was largely unrelated to accuracy estimates from the default classifiers (*p* values ≥ 0.249), except for a weak correlation between alpha and Logistic Regression model estimates [*r*_*s*_(10) = −0.515, *p* = 0.087].

All alpha models using default window parameters in the 1 Hz condition yielded mean test accuracies with a statistically higher median than chance (Uniform Random model, *Mdn* = 0.509; all *p* values ≤ 0.005). However, these relationships were much weaker during 4 Hz presentations, such that none of the three alpha classifiers were statistically distinguishable from the Uniform Random model (*Mdn* = 0.481; *p* values ≥ 0.06). Using individually tuned baseline and effect window latencies, all alpha models performed above chance (*Mdn* = 0.514) in the 1 Hz condition (all *p* values ≤ 0.005). In the 4 Hz condition however, Tangent Space (*Mdn* = 0.523) accuracies fell to the level of the Uniform Random model (*Mdn* = 0.501; *p* = 0.126), though the Logistic Regression (*Mdn* = 0.542) and Support Vector (*Mdn* = 0.533) models performed above chance (*p* values ≤ 0.050). Expectedly, tuned classifier mean test accuracies demonstrated higher median values for Logistic Regression models in both the 1 Hz (*p* = 0.045) and 4 Hz conditions (*p* = 0.005), relative to their default-parameter counterparts. Parameter tuning had no significant effect on the accuracy estimates of the Uniform Random, Support Vector, or Tangent Space models.

### Supplementary Analyses

Using tuned time-frequency analysis parameters, we reprocessed and reanalyzed the alpha data in BrainVision Analyzer in order to describe changes due to altered baseline and effect windows. Because tuning was performed using all four occipitoparietal channels, the supplementary across-participant analysis only utilized the pooled signal. Alpha activity distributions were normally distributed across participants. However, to remain consistent with our other analyses, median comparisons were again run using related-samples Wilcoxon signed-ranks tests. Median target (*Mdn* = −0.179) alpha activity was lower than median non-target activity (*Mdn* = 0.284) at 1 Hz presentations, and the same was true for targets (*Mdn* = 0.001) and non-targets (*Mdn* = 0.187) in the 4 Hz condition (both comparisons: *Z* = −2.589, *p* = 0.010). Alpha attenuation effects measured after parameter tuning did not correlate with any of the ERP target effects [all absolute *r*_*s*_(10) values ≤ 0.336; all *p* values ≥ 0.286].

Much like the within-participant alpha distributions discussed in sections “Alpha Effects: Within-Participants” and “Artifact Rejection,” distributions generated from individually tuned parameters remained non-normal in shape. Three previously significant alpha attenuation effects weakened to a trending state (*p* < 0.10), while another 3 were no longer significant following tuning procedures. However, two previously not-significant effects shifted to a trending state (note: one of these was in the opposite direction), while another became significant. Interestingly, the one effect that moved from not-significant to significant following tuning was observed in an individual with one of the weaker alpha attention effects, as measured in the primary analysis. Overall, following tuning procedures in the 1 Hz condition, 7/12 individuals demonstrated alpha attenuation in at least one signal source (6 at both pooled and Pz-only signals; 1 only in the pooled signal), with an additional 3 trending (2 in the pooled signal; 1 at Pz-only). One-half (6/12) of participants demonstrated alpha attenuation in at least one tuned signal source during the 4 Hz condition (1 in both sources; 3 only in the pooled signal; 2 at Pz-only).

## Discussion

The primary objectives of this exploratory study were (1) to detect target-related posterior alpha attenuation in a BCI RSVP paradigm, and (2) to discern whether that attenuation effect varied according to the presentation rate of the stimuli in that task. Secondarily, this study attempted to describe the relationship between changes in alpha and coincident ERP signals, and explore a subset of classification approaches to distinguish target and non-target alpha responses.

### Summary of Findings

As hypothesized, we observed significant event-related attenuation of posterior alpha activity across participants for target letter stimuli relative to non-targets. This alpha attenuation effect was observed in both 1 and 4 Hz presentation conditions, though as expected, target-related attenuation was significantly greater in the 1 Hz condition. Within-participant findings were similar, such that more individuals demonstrated target-related posterior alpha attenuation in the 1 Hz condition than in the 4 Hz condition. Significant target effects were observed for both N200 and P300 ERPs, but were unrelated to alpha attenuation. Classifier models trained to target/non-target classification of alpha activity performed above chance, but underperformed markedly relative to RDA/KDE estimates generated from ERP time-series data. Alpha attenuation effects were observed using Z-transforms based on pre-defined baseline and response windows. Individualized parameter tuning of these temporal windows resulted in improved performance of one of the alpha classifiers.

### The Disconnect Between Alpha Activity and Event-Related Potentials

In review, there is evidence that (1) posterior alpha and ERPs such as the P300 can capture similar attentional processes (Yordanova et al., 2001; Grent-’T-Jong et al., 2011), but also, that (2) alpha activity and attentional ERPs are not necessarily identical neural indices of attention (Klimesch, 1999; Peng et al., 2012). In agreement with these previous findings, the non-overlapping nature of alpha, N200, and P300 activity was apparent in the current study. Specifically, target-related fluctuations in alpha activity did not correspond to similar target-related changes in N200 or P300 signals across individuals.

### Individual Differences

Although posterior alpha attenuation was visible across-participants, within-participant analyses revealed clear individual differences in both the presence and size of the alpha attenuation effect. Individual differences in alpha activity are well-documented, including differences in IAF (Corcoran et al., 2018), as well as the amount and distribution of alpha activity (Bazanova and Vernon, 2014). With these differences in mind, it seems fair to question the absolute utility of posterior alpha for BCI target classification across all individuals. As shown in Figure 5, some participants demonstrated almost no attention-related differences in alpha activity, while a handful displayed consistent and pronounced effects. To be sure, this inter-individual variability is not a new difficulty in the field of BCI. User-centered design has long been an important topic (Akcakaya et al., 2014; Moghadamfalahi et al., 2015), and even some of the few lateralized posterior alpha designs have documented individual differences among users (Horschig et al., 2015). A viable approach to an alpha-compatible BCI likely includes identification of alpha “responders,” or individuals who demonstrate target-related changes in alpha activity, prior to including alpha activity as a classifiable signal in any machine learning model. To this point, a study from van Gerven and Jensen (2009) characterized different patterns of classification performance in participants sorted according to their “good” or “bad” alpha responses. Prior work has also demonstrated the benefits of personalizing channel selection to measure posterior alpha for BCI control (van Gerven et al., 2009).

### Electroencephalography Ensemble Methods

With high-dimensional data such as EEG, one alternative to classification using a single model is to instead use ensemble learning to combine multiple EEG classification approaches (Sun et al., 2007). Unlike multi-modal approaches that integrate different input modalities (e.g., EEG, EOG, EMG) to improve BCI function (Li et al., 2016), ensemble learning can deploy multiple analytic models for a single input. To this end, the current RSVP speller might be altered to consider an ensemble that simultaneously includes both ERP information and posterior alpha activity. Indeed, recent research has combined event-related frequency band information with ERP information for ensemble classification in a motor imagery task (Luo et al., 2020). A past investigation has also proposed that posterior alpha may be simultaneously useful as a marker of both attentional engagement and abrupt changes in mental state, including frustration (Myrden and Chau, 2017). Given the comparatively low performance of the alpha classifiers in this study, it seems prudent to explore integration of alpha and ERPs to improve classification of user intent in RSVP and other comparable paradigms.

## Limitations and Future Directions

Generalizability of this study is hindered by a small sample size (*n* = 12). Participants were generally healthy adults, so it remains to be seen whether our results extend to individuals with locked-in syndrome or other clinical populations. The use of pooled occipital sites for alpha and ERP analyses was relatively coarse, and future research would benefit from use of a recording apparatus of higher spatial resolution in order to evaluate topographic features of alpha attenuation in the context of RSVP. Artifact rejection methods resulted in only marginal changes to the data, possibly due to changes in statistical power after removing trials, since the across-participant measures did not change significantly following these rejections. Regardless, we recommend using these artifact rejection procedures consistently in future investigations. While overlapping processing of sequential visual stimuli is an inherent consequence of the RSVP design, the overlap was clearly detrimental to our measures of alpha activity in the 4 Hz condition. Additional work may benefit from attempts at noise reduction. A possible solution for this phenomenon would be to jitter stimulus duration to attenuate the SSVEP signal and associated harmonics. Lastly, though care was taken to match the preprocessing steps in both BrainVision and Python, there are minor differences inherent in the use of two different software packages. Future research would benefit from the use of a single, integrated toolbox.

## Conclusion

Event-related changes in posterior alpha activity are sensitive to visual attention during a common BCI speller paradigm, RSVP. Machine learning classifiers were able to discern target and non-target alpha responses at levels above chance, though these approaches were well below the current standards of more accepted ERP classifiers. However, the patterns of alpha attenuation observed in this study were not redundant when compared to ERPs, even though these signals capture similar aspects of visual attention. These findings also offer data on posterior alpha in a paradigm that differs from previous investigations which rely on covert spatial attention. For these reasons, posterior alpha activity is a viable candidate signal for inclusion in future BCI designs, most expectedly in ensemble with other EEG control measures.

## Data Availability Statement

The raw data supporting the conclusions of this article will be made available by the authors, without undue reservation.

## Ethics Statement

This study involving human participants was reviewed and approved by the OHSU IRB. The participants provided their written informed consent to participate in the study.

## Author Contributions

BO, DK, and TM designed the study protocol. DK and BO planned the statistical analysis. DK collected data, processed EEG recordings, performed statistical tests, and wrote the first draft of the manuscript. DE, NS-M, and BC designed the classification analysis. NS-M wrote the primary Python analysis script. NS-M, BC, and TM consulted on the code. TM, NS-M, and BC wrote portions of the manuscript. All authors contributed to revising the manuscript and approved the final draft.

## Conflict of Interest

The authors declare that the research was conducted in the absence of any commercial or financial relationships that could be construed as a potential conflict of interest.

## Publisher’s Note

All claims expressed in this article are solely those of the authors and do not necessarily represent those of their affiliated organizations, or those of the publisher, the editors and the reviewers. Any product that may be evaluated in this article, or claim that may be made by its manufacturer, is not guaranteed or endorsed by the publisher.
